# Long non-coding RNAs in anti-cancer drug resistance

**DOI:** 10.18632/oncotarget.12461

**Published:** 2016-10-04

**Authors:** Qin-nan Chen, Chen-chen Wei, Zhao-xia Wang, Ming Sun

**Affiliations:** ^1^ Department of Oncology, Second Affiliated Hospital, Nanjing Medical University, Nanjing, People's Republic of China; ^2^ Department of Bioinformatics and Computational Biology, UT MD Anderson Cancer Center, Houston, Texas, United States of America

**Keywords:** long non-coding RNAs, cancer, drug resistance, chemotherapy, targets

## Abstract

Chemotherapy is one of the basic treatments for cancers; however, drug resistance is mainly responsible for the failure of clinical treatment. The mechanism of drug resistance is complicated because of interaction among various factors including drug efflux, DNA damage repair, apoptosis and targets mutation. Long non-coding RNAs (lncRNAs) have been a focus of research in the field of bioscience, and the latest studies have revealed that lncRNAs play essential roles in drug resistance in breast cancer, gastric cancer and lung cancer, et al. Dysregulation of multiple targets and pathways by lncRNAs results in the occurrence of chemoresistance. In this review, we will discuss the mechanisms underlying lncRNA-mediated resistance to chemotherapy and the therapeutic potential of lncRNAs in future cancer treatment.

## INTRODUCTION

Malignant cancer is the most severe disease threatening human health, considerably reducing the quality of human lives. With the advent of various anticancer drugs, cytotoxic and molecularly targeted compounds have become the first-line standard treatment regimens for most cancer patients when surgery is not an appropriate option [1, 2]. In 2004, it was first reported that a mutation in the epidermal growth factor receptor (EGFR) conferred a clinical response to EGFR tyrosine kinase inhibitors (TKIs) in non-small cell lung cancer (NSCLC). To date, three generations of EGFR TKIs have been developed, and the third-generation molecular targeted drug WZ4002 exhibits high activity against tumor cells harboring EGFR with the T790M mutation. Clinical trials showed that progression-free survival (PFS) of patients with detectable T790M was significantly prolonged after taking the third-generation TKIs [3–5]. Human epidermal growth factor receptor-2(HER2) positive breast cancer accounts for about a quarter of breast cancers. HER2 amplification induces cell growth and suppresses cell death. Recently, the application of trastuzumab combined with chemotherapy was shown to increase overall survival (OS) of patients with HER2 overexpression by achieving a blockade of HER2 receptors [6]. Similarly, the combination of cisplatin and trastuzumab is the mainstream therapy for patients with HER2-positive gastric cancer [7]. The inhibition of EGFR2 and vascular endothelial growth factor receptor 2 have also confirmed their efficacy in the treatment of advanced gastric cancer [8]. Although chemotherapeutics have prolonged disease-free survival and OS for many patients, an inevitable problem that has gradually emerged is the propensity of tumor cells to become resistant to drugs that have been used previously, or even to drugs that are chemically and functionally unrelated, suggesting that tumor cells can adopt common resistance mechanisms [9]. Both intrinsic and acquired drug resistance can greatly limit the effectiveness of chemotherapy [10, 11]. Solving this problem is an urgent concern.

With the rapid development of bioinformatics analysis and application of next-generation sequencing technology to whole genomes and transcriptomes, it has become clear that only 2% of the human genome encodes proteins whereas 98% of transcriptional products are non-coding RNAs [12]. Most non-coding RNAs consist of more than 200 nucleotides, and are defined as long (or large) non-coding RNAs (lncRNAs) [13, 14]. For past decade, lncRNAs have been varified to participate in a series of cellular processes including cell proliferation, apoptosis, migration, and invasion and regulate gene expression at epigenetic, transcriptional, and post-transcriptional levels dependent on diverse cell locations. Importantly, a large amount of misregulated lncRNAs have been linked to human cancers development and progression. These lncRNAs involve in regulation of cancer cells growth, metastasis and chemotherapy drug resistance through diverse mechanisms, including interacting with RNA binding proteins such as polycomb repressive complex 2 (PRC2), behaving as decoys to compete with other proteins for the binding position of target genes or specific microRNAs, and modifying mRNA structure and affecting stability of mRNA [15–18]. Interestingly, many well-known transcription factors (such as E2F1, P53, SP1 et al.) and epigenetic regulators (such as EZH2, DNMT1) mediated DNA methylation or histone modifications have been found to contribute to lncRNAs aberrant transcriptional activation or inactivation in cancer cells [19–21]. In this review we describe multiple mechanisms of drug resistance including drug efflux, DNA damage repair, mutations of drug targets, and cancer cell apoptosis and highlight the important roles of long non-coding RNAs in the regulation of drug resistance of cancer cells.

## MECHANISMS OF CANCER CELL DRUG RESISTANCE

### Alterations in drug efflux

The ATP-binding cassette family in humans possesses 49 known transporters that move drug compounds out of cells to sustain intracellular drug concentration, directly leading to multidrug resistance (MDR) in cancer cells. Among these proteins, P glycoprotein (P-gp), adenosine triphosphate-binding cassette superfamily G member 2 (ABCG2), and multi-drug resistant associate protein (MRP) have been extensively studied in many solid tumors such as breast cancer and ovarian cancer [22]. P-gp is overexpressed in several cancers including neuroblastoma, myeloma, and colorectal cancer. It has been demonstrated that overexpression of P-gp predicts an unfavorable prognosis [23]. The expression of P-gp encoded by the MDR1 gene increases when normal tissues are transformed to a neoplastic state [24]. Some proteins, such as H-Ras, Raf-1, MEK1, and MEK2 involved in MAPK pathway, act as downstream receptors that upregulate the P-gp level, thus regulating the cellular environment and leading to the development of drug resistance. Conversely, inhibition of ERK pathway reduces P-gp expression [25, 26].

### Dysfunction of DNA damage repair

In normal cells, the DNA repair pathway is activated when DNA damage is induced by physical, chemical, or biological factors. To sustain the stabilization of chromosomes, DNA damage is efficiently repaired through activation of repair genes. Conversely, dysfunctional activation of the DNA repair pathway readily results in the occurrence of tumors [27]. It is well known that chemotherapeutics trigger DNA damage through direct or indirect mechanisms, which may contribute to the acquisition of cytotoxicity. If such damage can be repaired in tumor cells, there is a possibility that they will survive under chemotherapy or become more tolerant to chemotherapeutic agents. For instance, DNA is the key target of traditional chemotherapy drugs such as platinum, and cancer cells tend to be more resistant to platinum as a result of abnormal DNA damage repair activation [28, 29]. A latest study has observed that NF-κB/HOTAIR have interaction in DNA damage response in development of chemoresistance [30]. Accordingly, specific inhibition of DNA repair is believed to improve the efficacy of chemotherapeutics.

### Apoptosis

Two classic pathways are involved in cell apoptosis: the intrinsic pathway regulated by chondriosomes and the extrinsic pathway regulated by tumor necrosis factor (TNF) receptors. [10] The Bcl-2 protein family includes both apoptosis-inducing proteins such as Bax, Bad, and Bid, and antiapoptosis proteins like Bcl-2 and Bcl-xl. These proteins antagonize each other to maintain a relatively balanced condition in cells. Once the balance is disrupted, resistance to chemotherapy drugs that interefer apoptosis may arise during tumorigenesis [31]. It has been demonstrated that downregulation of Bcl-2 can increase sensitivity to chemotherapeutics [32]. In addition, high expression of Bcl-xl predicts poor prognosis in NSCLC [33]. Alterations in protein expression of TNF family members such as TNFR-1, Fas, DR4, and DR5 may lead to resistance to anticancer drugs. It has been noted that soluble Fas could block apoptosis induced by Fas [34, 35]. A clinical trial has verified that mutation of DR4 and DR5 contributes to drug resistance in glioma [36]. A recent study observed that expression of P-gp is inversely associated with expression of TNF-related apoptosis-inducing ligand or Apo2L (TRAIL), which mediates the apoptosis pathway in MDR cells. TRAIL has been implicated as a potent therapeutic target in clinical trials [37].

### Mutation of drug targets

Molecular targeted therapy is an advanced treatment option in cancer therapy that has become a major focus in cancer research because of fewer side effects and higher efficacy than standard chemotherapy agents [38]. Several different molecules can be considered targets for therapy, such as members of the signal transduction pathway; however, clinical tests have determined that drug resistance can be achieved when the pathway is altered [39–41]. Estrogen receptor (ER) positive breast cancer patients have a favorable prognosis compared with ER-negative patients but exhibit a higher recurrence rate following endocrine treatment. This is attributed to a decrease in ER-positive breast cancer cells. Moreover, activation of the PI3K/AKT/mTOR pathway results in resistance to endocrine drugs [42, 43]. Patients with lung cancer usually acquire resistance to EGFR TKIs due to the T790M mutation, the secondary mutation in EGFR [44]. Overexpression of BCR-ABL1 may account for the main mechanism of imatinib resistance. Clinically, patients with the BCR-ABL1 T315I mutation lose sensitivity to most second-generation TKIs [45].

## LONG NON-CODING RNA IN CANCER CELL DRUG RESISTANCE

Recently, numerous lines of evidence have indicated that lncRNA expression is widely altered in cancers and that lncRNAs participate in various aspects of tumorigenesis through inactivation of tumor suppressors or activation of oncogenes [46–48]. For example, expression of the lncRNA MALAT1 is decreased by treatment with S-adenosyl methionine (SAM) suggesting that epigenetic regulation of MALAT1 expression is predominantly through DNA methylation [49]. Our previous studies have revealed that the expression pattern of several lncRNAs, such as HOTAIR, SPRY4-IT1, BANCR, and PVT1, is altered in human NSCLC and gastric cancer. Among these, PVT1 is significantly upregulated in NSCLC tissues and cells, and increased PVT1 expression promotes NSCLC cell proliferation and suppresses apoptosis through epigenetic repression of transcription of the tumor suppressor LATS2 by binding with enhancer of zeste 2 polycomb repressive complex 2 subunit(EZH2) [50]. In addition, the lncRNA HOTAIR is overexpressed in gastric cancer and either promotes cell proliferation and metastasis by functioning as a ceRNA to sponge up miR-331-3p or epigenetically silences miR34a by binding to PRC2 [51, 52]. HOTAIR has also been identified as a cell proliferation regulator through binding to EZH2 in glioma cells [53]. Moreover, lncRNA BC032469 can directly bind to miR-1207-5p as a ceRNA that may decrease the expression of telomerase reverse transcriptase in gastric cancer, and UCA1 can sponge miR-485-5ps and antagonize its repression of matrix metallopeptidase 14 in epithelial ovarian cancer cells [54, 55]. Notably, emerging evidence has shown that lncRNAs are also actively involved in cancer cell drug resistance. Meijer et al. first identified BCAR4 through a functional genetic screen in the ER-positive and estrogen-dependent breast cancer cell line ZR-75-1. Ectopic expression of BCAR4 in ZR-75-1 cells induces hydroxytamoxifen resistance [56]. Moreover, knockdown of extracellular vesicle long non-coding RNA derived from extracellular vesicles reduced expression of ABCG2, promoting sorafenib-induced cell apoptosis in hepatocellular carcinoma [57]. PVT1 is associated with cisplatin resistance by inhibiting apoptotic pathways in ovarian cancer [58]. The lncRNAs involved in cancer cell drug resistance and their regulated targets/pathways related to cancer drug resistance are listed in Table 1.

**Table 1:** Cancer drug resistance related lncRNAs

lncRNAs	Targets	Mechanisms	Drugs	Cancers	Refs
LEIGC	N/A	N/A	5-Fu	Gastric cancer	[58]
MRUL	ABCB1	N/A	Multi-drug resistance	Gastric cancer	[59]
AK022798	MRP1, P-glycoprotein	N/A	Cisplatin	Gastric cancer	[60]
PVT1	MDR1, MRP1	N/A	Multi-drug resistance	Gastric cancer	[61]
	N/A	N/A	Cisplatin	Ovarian cancer	[47]
ANRIL	PARP, bcl-2	N/A	Cisplatin, 5-Fu	Gastric cancer	[63]
HOTAIR	GREB1, TFF1, c-MYC	N/A	Tamoxifen	Breast cancer	[52]
	P21	N/A	Cisplatin	NSCLC	[72]
	IL-6	Activating NF-κB signaling	Platinum	Ovarian cancer	[20]
	HOXA1	DNA methylation	Multi-drug resistance	SCLC	[77]
LncRNA-ATB	miR-200c	CeRNA	Trastuzumab	Breast cancer	[53]
BCAR4	ERBB2/ERBB3	N/A	Oestrogen, Tamoxifen	Breast cancer	[48–51]
HIF1A-AS2	N/A	N/A	Paclitaxel	Breast cancer	[55]
AK124454	N/A	N/A	Paclitaxel	Breast cancer	[55]
UCA1	Wnt6	N/A	Cisplatin	Bladder cancer	[67]
	PARP, bcl-2	N/A	Adriamycin	Gastric cancer	[62]
	miR-204-5p	CeRNA	5-Fu	Colorectal cancer	[84]
AK126698	NKD2	N/A	Cisplatin	NSCLC	[74]
GAS5	IGF-1R	N/A	Gefitinib	NSCLC	[75]
	miR-21	CeRNA	Trastuzumab	Breast cancer	[54]
MEG3	P53, bcl-xl	N/A	Cisplatin	NSCLC	[73]
LINC00635-001	Akt	N/A	Gefitinib	NSCLC	[76]
ODRUL	ACBC1	N/A	Doxorubicin	Osteosarcoma	[78]
H19	MDR1, P-glycoprotein	Binding with DNA methyltransferases	Doxorubicin	Hepatocellular cancer	[80]
linc-ROR	CD133	N/A	Sorafenib, doxorubicin	Hepatocellular cancer	[81]
CCAL	AP-2α, MDR1, P-glycoprotein	activating Wnt/β-catenin pathway	Multi-drug resistance	Colorectal cancer	[83]
snaR	N/A	N/A	5-Fu	Colorectal cancer	[82]
HOTTIP	HOXA13	N/A	Gemcitabine	Pancreatic cancer	[79]
lncARSR	miR-34/miR449	CeRNA	Sunitimb	Renal cancer	[85]

ABCB1,ATP binding cassette subfamily B member 1; MRP1, multi-drug resistant associate protein 1; MDR1, multi-drug resistant protein; ceRNA, competing endogenous RNA; 5-Fu, 5-fluorouracil; NSCLC, non-small cell lung cancer; N/A, not available.

### lncRNAs and breast cancer drug resistance

**Figure 1:**
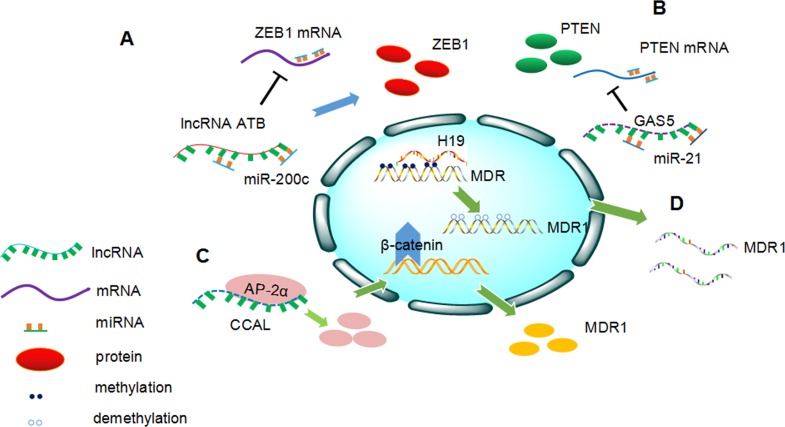
Overview of the involvement of long non-coding RNAs (lncRNAs) in cancer drug resistance **A.** LncRNA-ATB competitively sponge miR-200c, downregulating ZEB1 expression, thus inducing trastuzumab resistance in breast cancer. **B.** GAS5 suppress the expression of phosphatase and tensin homologs (PTEN) by sponging miR-21. Downregulation of GAS5 leads to trastuzumab resistance in breast cancer. **C.** CCAL promotes MDR1 expression through activating Wnt/β-catenin pathway by targeting AP-2α in colorectal cancer. **D.** H19 induces MDR1 expression *via* increasing the MDR1 promoter methylation level in hepatocellular carcinoma.

ER-positive mammary cancers are mostly dependent on estrogenic growth stimulation and antihormone therapy is the major clinical treatment for ER-positive breast cancer. Regrettably, although anti-hormone therapy is widely applied to cure breast cancer patients, it cannot totally suppress the growth of breast cancer cells [59]. BCAR4 is a strong oncogene that transforms breast cancer cells into an estrogen-independent, antiestrogen-resistant state. In addition, loss of estrogen receptor 1(ESR1) does not result in tamoxifen resistance, as verified in ZR/BCAR4 cells, and inhibition of ESR1 does not affect the drug resistant capacity of ZR/BCAR4 cells [60]. In 2010, Godinho et al. showed that high levels of BCAR4 predict poor PFS and patients with high expression of BCAR4 are likely to be resistant to endocrine therapy. In addition, expression of ERBB2 and ERBB3 was elevated in ZR/BCAR4 cells, indicating that activation of ERBB2/ERBB3 signaling may contribute to BCAR4-induced proliferation in the presence of tamoxifen. Cell proliferation was inhibited after knockdown of BCAR4. An identical result was observed upon knockdown of ERBB2/3, implying that BCAR4 acts in an ERBB2/3-dependent manner [59, 61, 62]. Recently, Xue et al. found that HOTAIR expression was significantly higher in tamoxifen-resistant breast cancer tissues compared with primary cancer tissues. HOTAIR was directly repressed by estrogen and conversely upregulated in the absence of hormone. Interestingly, increased HOTAIR expression may strengthen ER signaling, stimulating ER transcriptional activities even under an estrogen-deprived environment. Moreover, functional studies revealed that a high level of HOTAIR promoted the growth of breast cancer, whereas silencing of HOTAIR abolished tamoxifen-resistant cell growth [63]. Shi et al. found that lnc-ATB was remarkably upregulated in trastuzumab-resistant breast cancer cells and tissues. Lnc-ATB could promote trastuzumab resistance and then induce an invasion-metastasis cascade in breast cancer by competitively sponging miR-200c, thereby upregulating ZEB1 and ZNF-217. In addition, overexpression of lnc-ATB was positively associated with trastuzumab resistance of breast cancer patients(Figure 1) [64]. Li et al. reported that GAS5 suppressed cancer cell growth by sponging miR-21, resulting in downregulation of phosphatase and tensin homolog(PTEN), the target of miR-21(Figure 1) [65]. Triple-negative breast cancer account for one fifth of all breast cancers. Jiang et al reported lncRNA HIF1A-AS2 and AK124454 contributed to paclitaxel resistance in triple-negative breast cancer through transcriptome analysis [66].

Together, these findings provide new insight into breast cancethe involvement of lncRNAs in breast cancer drug resistance, and it is essential to identify more lncRNAs that could be potential therapeutic targets for chemotherapy-resistant breast cancer patients [67].

### lncRNAs in gastric cancer drug resistance

Several studies have documented that various lncRNAs are dysregulated in gastric cancer, and that their aberrant expression is related to tumorigenesis, metastasis, or drug resistance. Han et al. found that LEIGC knockdown in MGC-803 cells resulted in reduced sensitivity of gastric cancer cells to 5-fluorouracil (5-FU) [68, 69]. Wang et al. showed that the lncRNA MRUL was located near the MDR1 gene region and that expression of MRUL was higher in both SGC7901/VCR and SGC7901/ADR cells than in SGC7901 cells. P-gp-related chemotherapy drugs are considered to be the standard treatment for patients encountering MDR. Patients with high MRUL levels responded negatively to chemotherapy drugs. Consistent with this finding, downregulation of MRUL enhanced chemosensitivity of MDR gastric cancer cell sublines to P-gp-related chemotherapy drugs. MRUL knockdown in MDR cells led to increased doxurubicin concentration and a reduced Bcl-2/Bax ratio that may promote the rate of apoptosis. Additionally, *in vitro* and *in vivo* results showed that MRUL depletion decreased ATP binding cassette subfamily B member 1 (ABCB1) mRNA levels. Heterologous luciferase reporter assays showed that MRUL performed an enhancer-like role to promote ABCB1 transcription [70]. Hang et al. found that Notch 1 overexpression positively regulated lncRNA AK022798 during gastric cancer progression. Silencing of AK022798 significantly reduced the cell viability of cisplatin-resistant cell lines SGC7901/DDP and BGC823/DDP and the expression of MRP1 and P-gp, and increased apoptosis of SGC7901/DDP and BGC823/DDP cells. AK022798 may become a new target for the treatment of terminal-stage gastric cancer [71]. Zhang et al. reported that PVT-1 was highly expressed in gastric cancer tissues of cisplatin-resistant patients and BGC823/DDP and SGC7901/DDP cells. In addition, transfection of BGC823/DDP and SGC7901/DDP cells with PVT-1 siRNA could overcome the resistance of these two cisplatin-resistant cell lines, whereas overexpression of PVT1 exhibited antiapoptotic activity in BGC823 and SGC7901 cells exposed to cisplatin. Moreover, qRT-PCR and western blotting analyses showed that the expression of MDR1, MRP, mTOR, and HIF-1a increased upon upregulation of PVT1. These findings suggest that lncRNA PVT1 may play a critical role in the development of MDR in gastric cancer [72]. Shang et al revealed that UCA1 knockdown inhibited the resistance to adriamycin of SGC7901/ADR cells, UCA1 silencing promoted apoptosis through upregulating expression of PARP and suppressing Bcl-2 levels [73]. Lan et al found that ANRIL was greatly upregulated in cisplatin resistant and 5-Fu resistant patients. The rate of tumor growth significantly decreased after transfected with si-ANRIL, and the levels of MDR1, MRP1 also reduced [74].

### lncRNAs in bladder cancer drug resistance

Platinum-based chemotherapy is the standard first-line treatment for bladder cancer, whereas gemcitabine plus cisplatin is approved for metastatic urothelial cancer. However, most patients ultimately experience disease recurrence due to the poor response to therapy [75]. Wang et al. used RACE technology to obtain full-length cDNA for UCA1, which is believed to play a role in bladder cancer progression. Cell viability studies by MTT assay showed that expression of UCA1 in BLS-211 cells caused resistance to cisplatin, and further studies determined that the level of serine-arginine protein kinase 1 was inversely related to UCA1 expression [76]. Wang et al. reported that overexpression of UCA1a led to fewer apoptotic cells after cisplatin treatment [77]. Fan et al. suggested that upregulation of UCA1 in patients with bladder cancer partially contributed to cisplatin-based therapy. Likewise, UCA1 expression levels were higher in cisplatin-resistant bladder cancer cells. Forced expression of UCA1 augmented cell viability even in the presence of cisplatin, whereas UCA1 inhibition reduced cell viability during cisplatin treatment. Furthermore, UCA1 remarkably increased expression of Wnt6 in human bladder cancer cell lines, and their expression was also positively correlated *in vivo*. Finally, UCA1 promoted cisplatin resistance of bladder cancer cells by enhancing the expression of Wnt6 and activating Wnt signaling. Thus, the UCA1/Wnt6 pathway represents a potential target for conquering chemoresistance in bladder cancer [78].

### lncRNAs in lung cancer drug resistance

The developments of platinum-based chemotherapy and targeted therapies for EGFR-sensitive and ALK-positive patients have been milestones in lung cancer treatment [79]. Nonetheless, increasing proportions of patients eventually develop acquired resistance [80]. Cheng et al. speculated that lncRNAs may play a pivotal role in resistance to EGFR-TKIs. They found that numerous lncRNAs were differentially expressed in gefitinib-sensitive and gefitinib-resistant cells using lncRNA microarray. Bioinformatics analysis showed that these aberrantly expressed lncRNAs were involved in regulating resistance to EGFR-TKIs by influencing neighboring genes. Pathway analysis revealed that cell proliferation and apoptosis were associated with the development of EGFR-TKI resistance [81]. In addition, Wu et al screened 1476 lncRNAs dysregulated in EGFR-TKI-resistant cell line of lung adenocarcinoma, which further illustrated lncRNAs may play as biomarkers in EGFR-TKI therapy [82]. In our previous studies, we found that HOTAIR expression was significantly increased in cisplatin-resistant A549/DDP cells, and that siRNA-mediated silencing of HOTAIR could partly restore the responses of A549/DDP cells to cisplatin. Functional analysis demonstrated that p21 is the underlying target of HOTAIR and overexpression of p21 partially rescued the HOTAIR-induced cisplatin resistance in A549/DDP cells [83]. In addition, we also revealed that MEG3 expression is decreased in A549/DDP cells, and exogenic overexpression of MEG3 partially reversed the cisplatin resistance of A549/DDP cells through the regulation of p53 and Bcl-xl expression [84]. Moreover, Yang et al. identified eight lncRNAs that were differentially expressed in A549/DDP cells. Downregulation of one of these lncRNAs—lincAK126698 depressed the induction of apoptosis by cisplatin in A549 cells, possibly through decreased naked cuticle homolog 2 expression and increased β-catenin expression resulting in altered Wnt signaling [85]. Dong et al. reported that a high level of GAS5 reduced tumor growth both *in vitro* and *in vivo* under treatment with gefitinib. In addition, they confirmed that IGF-1R is a key downstream mediator that was inversely correlated with expression of GAS5 [86]. Wu et al. demonstrated that linc00635-001 silencing accompanied by gefitinib treatment suppressed Akt activation and sensitized HCC827-8-1 cells to gefitinib-induced cytotoxicity [87]. Fang et al testified HOTAIR recruited HOXA1 by RNA immunoprecipitation. HOTAIR silencing reduced methylation of HOXA1, and enhanced the sensitivity of cancer cells to anticancer drugs in SCLC [88].

### lncRNAs in drug resistance of other cancers

Researchers demonstrated that expression of lncRNA ODRUL was increased in doxorubicin-resistant osteosarcoma cell lines. ODRUL knockdown led to suppression of the ABCB1 gene, which is related to multidrug resistance [89]. Silencing of HOTTIP increased the chemosensitivity of pancreatic cancer cells to gemcitabine. The expression of HOTTIP showed a positive correlation with HOXA13 and the biological behavior of HOTTIP was partially modulated by HOXA13. Moreover, a high level of HOXA13 predicted poorer prognosis in pancreatic cancer [90]. Tsang et al. observed that H19 inhibition decreased the expression of MDR1/P-glycoprotein and increased cellular doxorubicin accumulation and doxorubicin sensitization in both HepG2 parent cells and R-HepG2 cells. MDR1 promoter methylation was inversely correlated to MDR1 expression level, and only half of the CpG island sites at the MDR1 promoter region were hypomethylated in R-HepG2 cells. Furthermore, there was an increase in MDR1 promoter methylation level after H19 knockdown. These findings demonstrated that H19 altered P-glycoprotein expression and induced MDR1-associated drug resistance by modulating MDR1 promoter methylation (Figure 1) [91]. A recent study reported that linc-ROR expression was induced by sorafenib in HCC cells, whereas knockdown of linc-ROR enhanced chemotherapy-induced cell death. Silencing of linc-ROR attenuated the expression of the CD133+ cells present among tumor-initiating cells that resulted in progression of chemoresistance [92]. Lee et al. identified that upregulation of lncRNA snaR promoted apoptosis of colon cancer cells after 5-FU treatment. In contrast, loss of snaR decreased sensitivity of cancer cells to 5-FU [93]. Ma et al. showed that the lncRNA CCAL acted as an oncogene in colorectal cancer progression; patients with high CCAL expression had shorter survival and worse response to chemotherapy. CCAL mediated a reduction in AP-2α protein-activated Wnt/β-catenin signaling, inducing multidrug resistance and upregulating MDR1/P-gp expression. Moreover, CCAL was upregulated by histone H3 methylation and deacetylation in colorectal cancer (Figure 1) [94]. Bian et al detected that UCA1reduced sensitivity of colorectal cancer cells. UCA1 sponged miR-204-5p, activating CREB1 expression, which correlated with poor prognosis of patients [95]. Qu et fal found that exosome transmitted lnc ARSR functioned as ceRNA sponging miR-34/miR449 that may promote the expression of AXL and c-MET, thus inducing sunitinib resistance in renal cancer. It is believed that lncARSR or AXL/c-MET inhibitors may have curable potential in renal cancer treatment [96]. Özeş et al conducted that HOTAIR was highly expressed in ovarian cancer patients resistant to platinum therapy. The ectopic expression of HOTAIR could persistently repair DNA damage attributed by platinum, and it activated NF-κB signaling. It is suggested that NF-κB/HOTAIR crosslinking contributed to chemoresistance in ovarian cancers [30].

## FUTURE PERSPECTIVES

Cancer is a major cause of human disease-related death worldwide. Chemotherapy is one of the main treatment methods for cancer patients, and many newly developed molecular targeted drugs significantly improve therapeutic efficacy and prolonged patient survival time. However, resistance to chemotherapy drugs has become the most urgent problem hampering the treatment of cancer patients. Over past decades, substantial efforts have been devoted to the investigation of resistance mechanisms of cancer cells and approaches to reverse such resistance. Researchers have found that many protein coding genes such as MDR1, ABCG2, and MRP play critical roles in cancer cell drug resistance, and some of them have been used to develop treatment strategies for patients. In addition, many noncoding RNAs, including microRNAs and lncRNAs, have also been shown to be involved in this process [97–99]. Chen et al. reported that low expression of miR-206 correlates with restored cisplatin resistance in lung adenocarcinoma tissues, and that miR-206 suppresses cisplatin resistance *via* inactivating PI3K/AKT /mTOR signaling pathways [100]. Sun et al. proposed that upregulation of miR-424 and miR-27a enhanced TRAIL sensitivity by downregulating PLAG1 [101]. Besides, lncRNAs have been reported to act as oncogenes or tumor suppressor genes that can reduce or increase the sensitivity of cancer cells to anticancer regimens such as tamoxifen, gefitinib, cisplatin, docetaxel, and 5-Fu. More further studies are needed to determine whether lncRNA-based cancer therapy can be applied to clinical practice [102]. Although studies on lncRNA and cancer drug resistance remain in their infancy, we cannot ignore the potential of lncRNAs as candidates to develop novel strategies to reverse the cancer cell resistance to chemotherapy or molecular targeted therapy. Therefore, more researches are needed to identify additional lncRNAs related to cancer cell drug resistance and elucidate their function and molecular mechanisms, which may place lncRNAs at center stage in the biology of drug resistance of cancer cells.

## Abbreviations

EGFR: lncRNAs, long non-coding RNAs; epidermal growth factor receptor
TKIs: tyrosine kinase inhibitors
NSCLC: non-small cell lung cancer
PFS: progression-free survival
OS: overall survival
HER2: Human epidermal growth factor receptor-2
PRC2: Polycomb repressive complex 2
MDR: multidrug resistance
P-gp: P glycoprotein
ABCG2: Adenosine triphosphate-binding cassette superfamily G member 2
MRP: Multi-drug resistant associate protein
TNF: Tumor Necrosis Factor
TRAIL: TNF-related apoptosis-inducing ligand or Apo2L
ER: estrogen receptor
ceRNA: competing endogenous RNA
SAM: S-adenosyl methionine
EZH2: enhancer of zeste 2 polycomb repressive complex 2 subunit
ESR1: estrogen receptor 1
PTEN: phosphatase and tensin homolog
5-FU: 5-fluorouracil
ABCB1: ATP binding cassette subfamily B member 1
